# PDL1 expression and its correlation with outcomes in non-metastatic triple-negative breast cancer (TNBC)

**DOI:** 10.3332/ecancer.2021.1217

**Published:** 2021-04-06

**Authors:** Joydeep Ghosh, Meheli Chatterjee, Sandip Ganguly, Anupurva Datta, Bivas Biswas, Geetashree Mukherjee, Sanjit Agarwal, Rosina Ahmed, Sanjoy Chatterjee, Deepak Dabkara

**Affiliations:** 1 Department of Medical Oncology, Tata Medical Center, 14 MAR (E-W), New Town, Rajarhat, Kolkata, West Bengal 700156, India; 2 Department of Pathology, Tata Medical Center, 14 MAR (E-W), New Town, Rajarhat, Kolkata, West Bengal 700156, India; 3 Department of Breast Oncology, Tata Medical Center, 14 MAR (E-W), New Town, Rajarhat, Kolkata, West Bengal 700156, India; 4 Department of Radiation Oncology, Tata Medical Center, 14 MAR (E-W), New Town, Rajarhat, Kolkata, West Bengal 700156, India

**Keywords:** PDL1, early breast cancer, triple negative

## Abstract

**Purpose:**

Triple-negative breast cancer (TNBC) has a poor outcome compared to other subtypes, even in those with early disease. Immune checkpoint inhibitors (ICIs) have been approved in metastatic diseases and are being tested as a neoadjuvant strategy also. The response to ICIs is largely determined by the ﻿programmed death ligand 1 (PDL1) score, which also acts as a prognostic marker for outcomes. Here, we report the proportion of PDL1 expression in ﻿non-metastatic TNBC and its correlation with response to chemotherapy and outcomes.

**Methods:**

We included all patients who had non-metastatic TNBC treated with neoadjuvant chemotherapy, followed by surgery with/without adjuvant radiotherapy between September 2011 and November 2017. PDL1 testing was carried out on pre-treatment tumour cells with immunohistochemistry (Ventana SP142) and was correlated with pathological response, relapse-free survival (RFS) and overall survival (OS). PDL1 staining was interpreted as negative or positive (more than 1% staining).

**Results:**

A total of 107 patients were included for analysis with a median age of 47 years (28–65 yrs). The PDL1 expression of more than 1% was seen in 31 (28.97%) patients. After a median follow-up of 55 months (range: 4–93 months), median RFS and OS were not reached. PDL1 expression did not affect the achievement of pathological complete response (pCR). However, PDL1 expression improved OS (*p* = 0.016) and trend towards RFS (*p* = 0.05). Patients who achieved pCR had better RFS and OC compared to those who did not.

**Conclusion:**

Our study shows PDL1 expression in 29% of the cases. PDL1 expression leads to better RFS and OS. Also, pCR improves survival.

## Abbreviations

PDL1: programmed death ligand 1, TNBC: triple-negative breast cancer, FEC-T: 5-fluorouracil, epirubicin, cyclophosphamide and docetaxel, EC: epirubicin and cyclophosphamide, AC: adriamycin and cyclophosphamide, FEC: 5-fluorouracil, epirubicin and cyclophosphamide, FAC: 5-fluorouracil, adriamycin and cyclophosphamide, NACT: neoadjuvant chemotherapy, pCR: pathological complete response , NIH: National Institute of Health, PARP: poly(ADP-ribose)polymerase, RFS: relapse-free survival, OS: overall survival, BCS: breast conservation surgery, AD: axillary dissection, NLR: neutrophil lymphocyte ratio, PLR: platelet lymphocyte ratio, HER2: human epidermal growth factor receptor 2, IQR: interquartile range.

## Introduction

Triple-negative breast cancer (TNBC) is characterised by the absence of oestrogen receptors, progesterone receptors, and human epidermal growth factor receptor 2 (HER2) overexpression, usually in combination with a high proliferation index. It occurs more often in young women and patients with the BRCA1 mutation [1] (immune checkpoint blockade in patients with triple‑negative breast cancer) [2]. TNBC comprises 15%–20% of the total breast cancer cases globally [3]. Data from automated immunohistochemistry (IHC) analysis centres in India have reported a lower incidence (11%–12%) of TNBC subtype [4]. The outcomes of TNBC are far inferior to other subtypes [5, 6], despite having a higher response rate after neoadjuvant chemotherapy (NACT) [7, 8]. The response rate is further improved by the addition of carboplatin and recently poly(ADP-ribose)polymerase (PARP) inhibitors compared to standard anthracyclines and taxanes [9, 10]. Pathological complete responses (pCR) of TNBC correlate well with better relapse-free survival (RFS) and overall survival (OS) [11]. Moreover, tumours with a high degree of infiltrating lymphocytes (TILs) have a higher probability of achieving a pCR [12]. Immune checkpoint inhibitors (ICIs) such as atezolizumab have shown better outcomes when combined with chemotherapy in metastatic TNBC, more so in the programmed death ligand 1 (PDL1)-positive subset [13, 14]. Pembrolizumab, another ICI, has also been studied in the neoadjuvant setting and has showed significantly higher pCR [15]. All these studies have shown that ICIs’ benefit is more pronounced with higher PDL1 expression compared to those who had PDL1-negative tumours. Patients who did not have PDL1 expression also benefitted [13, 15]. PDL1 positivity in patients with TNBC is around 50% and is associated with better RFS [16]. The above-mentioned studies underline the importance of carrying out PDL1 for both prognostic and predictive factors. Studies in breast cancer have shown that there is a correlation between neutrophil lymphocyte ratio (NLR) and platelet lymphocyte ratio with long-term outcomes [17–20]. There is no data on the proportion of PDL1 expression in non-metastatic TNBC in the Indian subcontinent, and here we have analysed the PDL1 expression in those patients who received NACT, and also correlated it with pCR and survival outcomes.

## Aims

The primary aim of this study was to look at the proportion of non-metastatic TNBCs which express PDL1 in their tumour cells. The secondary aim was to look at the correlation of PDL1 with response (pCR) to NACT and its effect on long-term outcomes, the effect of pCR, neutrophil lymphocyte ratio and platelet lymphocyte ratio on pathological response and outcomes. The endpoints for measuring response are the presence or absence of pCR and for long-term outcomes are RFS and OS.

## Methods

### Patient information

**Figure 1.**
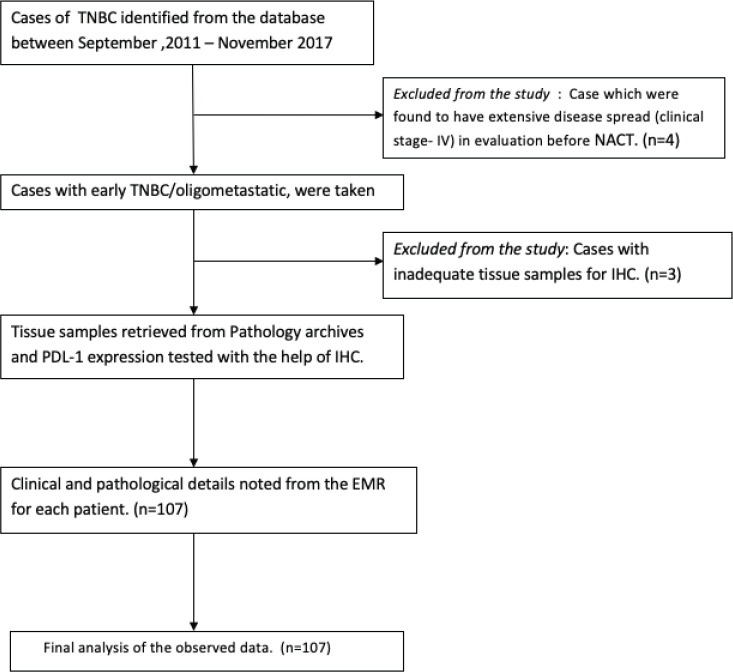
Consort diagram.

A consecutive series of patients was identified from retrospective analysis of pathology reports from electronic medical records (EMR). All those who had a diagnosis of non-metastatic TNBC who received NACT and then underwent breast surgery in our institution were included from June 2012 to June 2017. After initial histopathological confirmation and metastatic workup with contrast-enhanced computerised tomographic scan of thorax and whole abdomen and a bone scan, all of them received NACT containing anthracycline with/without taxane. After NACT, they underwent either mastectomy (MRM) or breast conservation surgery (BCS), and all of them had axillary dissection. All the cases were examined and reviewed by the breast pathology team. Pathological details including tumour size, nodal stage, grade, oestrogen receptor (OR), progesterone receptor (PR), and HER-2 score, tumour margin status and Ki-67 index were noted. The proportion of pCR, baseline neutrophil, lymphocyte count and platelet count was recorded. The median cut-off value for high NLR was taken as 3.0 and for platelet lymphocyte ratio (PLR) it was 185 based on a published meta-analysis [14, 15]. The pCR was defined either by the complete absence from both breast tissue and nodes or only the presence of *in situ* components [16]. The data on treatment received and follow-up were derived from the EMR. Male breast cancer was excluded from this study (Figure 1). Ethical clearance was obtained from the institutional review board and a waiver of consent was obtained in view of the non-interventional and retrospective nature of the study (EC/TMC/86/17).

### PDL1 testing

The PDL1 expression in the tissue was studied in tumour cells using immunohistochemistry. PDL1 testing was carried out in tumour cells and not the immune cells because at the time when this study was conducted there was no clear guideline on the role of immune cell PDL1 positivity or specific scoring methodology in breast cancer. Consecutive full-face 5-µm formalin-fixed paraffin-embedded sections were stained with PDL1 antibody (Ventana SP142) using the standard and validated protocol [21, 22]. Citrate low pH antigen retrieval was used for 20 minutes with the Bond III. PDL1 expression was assessed only in pre-therapeutic core needle biopsy specimens. All stained slides of pre-treatment biopsy were reviewed and scored by a single pathologist. PDL1 staining was evaluated in the tumour cells of the initial specimen of core biopsy before any chemotherapy, and not the surgical specimen. PDL1 staining 1% or more was considered positive. The PDL1 status was correlated with pCR, RFS and OS.

### Statistical analysis

Descriptive analysis was used for baseline clinico-pathologic features. OS was defined as the length of the time interval from the date of surgery to the date of the last contact or death. Relapse-free survival (RFS) was defined as the time interval from the date of surgery to the date of diagnosis of local and/or distant recurrence. RFS included both distant recurrences (metastasis to other organs not including regional lymph nodes) and local recurrences. The correlation between PDL1 expression and pCR with baseline parameters was assessed by univariate regression. Those that were found to be significant were considered for multivariate analysis. The survival was estimated with the Kaplan–Meier method. The predictors of survival were analysed by the Cox proportional model. Log rank tests were used to calculate the significance between survival functions. STATA (version 14.1, StataCorp, 4905 Lakeway Dr College Station, TX 77845) statistical software was used for statistical analysis [23].

## Results

### Clinico-pathologic features

A total of 107 patients were enrolled with a median age of 47 years (range: 28–65 years). Baseline clinico-pathologic features are mentioned in Table 1. Postmenopausal were 59 (55.14%) patients. Clinically relevant family history was seen in seven (6.6%) patients. Clinical stage distribution as per AJCC was Stage I = 1 (0.9%), Stage II = 38 (35.51%) and Stage III = 68 (63.55%). The median NLR was 2.85 (interquartile range (IQR): 2.15–4.22) and median PLR was 128 (IQR: 79.84–165.61). All the six cycles of 5-flurouracil, epirubicin, cyclophosphamide and docetaxel (FEC-T) regimen was given to 98 (91.6%) patients. Breast conservation surgery (BCS) was carried out in 52 (48.6%) patients. All patients received adjuvant radiotherapy. The mean number of nodes dissected was 21 (range: 1–37), and the mean number of nodes which were positive were 2 (range: 0–31).

Pathological complete response was observed in 35 (32.71%) patients. PDL1 expression more than 1% was seen in 31 (28.97%) patients, negative in 68 (63.55%) patients and non-interpretable in 8 (7.48%) patients.

### Response to chemotherapy

On univariate analysis, only the type of surgery (BCS versus MRM) correlated significantly with pCR (*p* < 0.001), with a greater number of patients in the BCS group who had pCR, as shown in Table 2. No other factors had a significant correlation with PDL1 expression, clinical stage, menopausal status, number of NACT cycles, NLR or PLR. On multivariate analysis, the statistical significance of the type of surgery (BCS versus MRM) persisted (*p* = 0.02), favouring BCS (Table 3).

### Survival outcome and prognostic features

**Figure 2.**
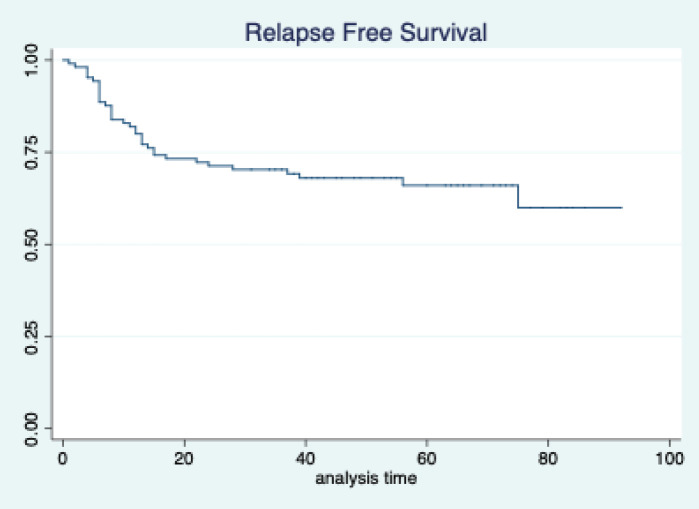
Relapse free survival of all patients.

**Figure 3.**
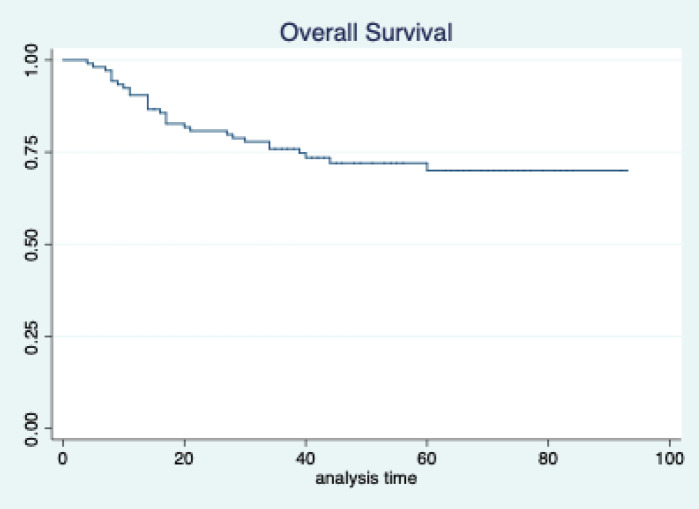
Overall survival of all patients.

**Figure 4.**
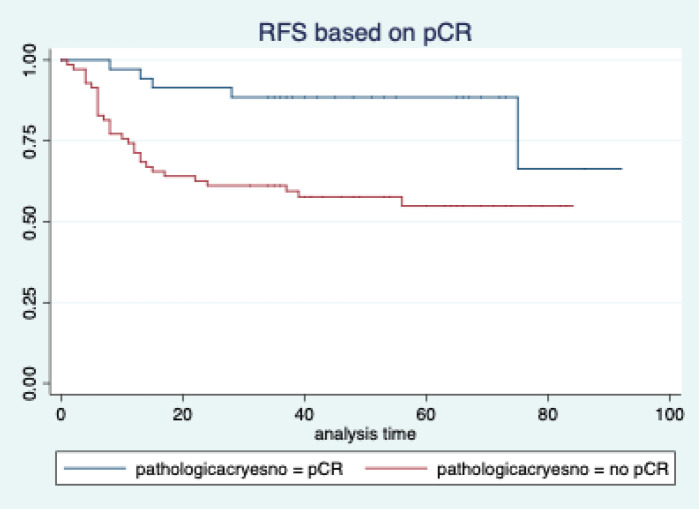
Relapse free survival based on pathological complete response.

**Figure 5.**
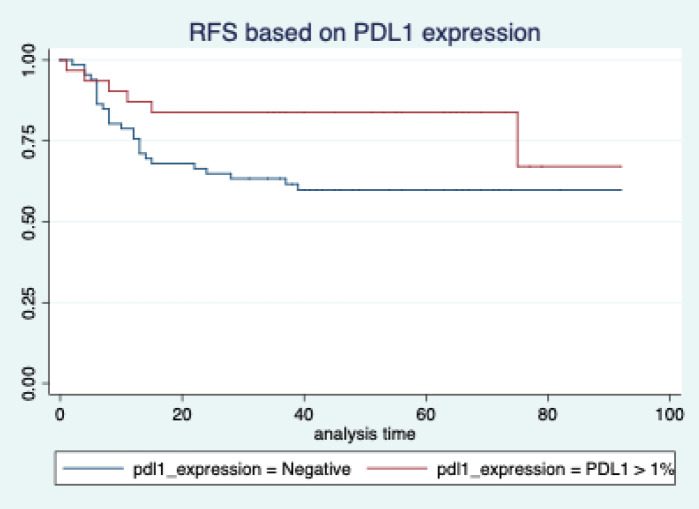
Relapse free survival based on PDL1 expression.

**Figure 6.**
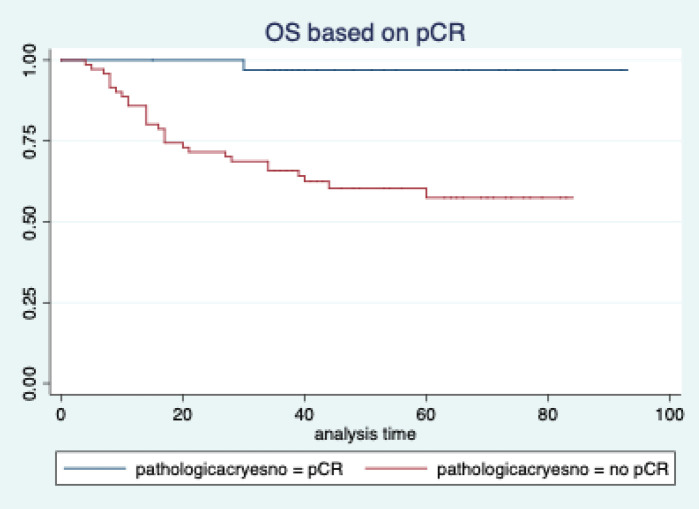
Overall survival based on pathological complete response.

**Figure 7.**
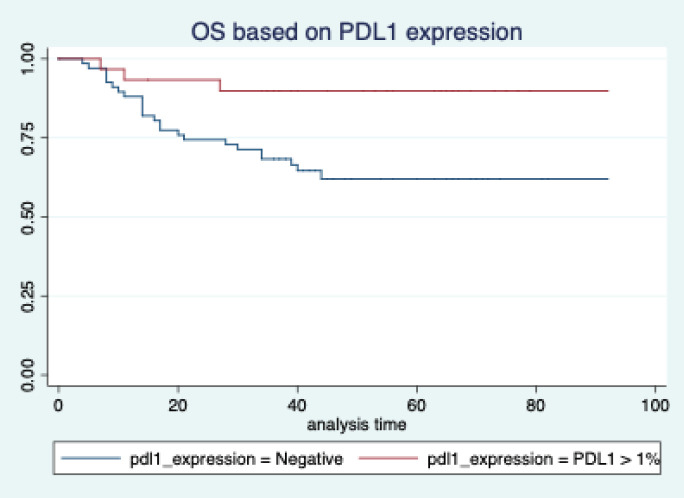
Overall survival based on PDL1 expression.

The median follow-up of patients included in this study was 55 months (range: 4–93 months). The median RFS and OS were not reached in this period (Figures 2 and 3). The achievement of pCR statistically and significantly improved the RFS (HR: 3.73, 95% CI: 1.44–9.63, p = 0.006) and the OS (HR = 16.46, 95% CI: 2.23–121.0, *p* = 0.006) compared to those who did not achieve pCR (Figures 4 and 5). The expression of PDL1 showed a trend towards improvement in RFS (HR: 0.41, 95% CI: 0.16–1.01, *p* = 0.055), but with OS, it was statistically significant (HR: 0.24, 95% CI: 0.07–0.82, *p* = 0.02) (Figures 6 and 7). Cox proportional-hazards for RFS and OS is shown in Table 4. None of the baseline parameters other than PDL1 and pCR had any significant impact on the RFS and OS. The differences between RFS and OS based on the NLR and PLR were non-significant (not reached in any group, HR: 0.78, 95% CI: 0.37–1.67, *p* = 0.527).

## Discussion

Our study contributes to the ongoing knowledge about the role of PDL1 affecting the outcomes of TNBC when they are treated with chemotherapy. High PDL1 may allow room for chemo de-escalation studies with addition of immune-oncology agents.

The median age of our patient population was 47 years. This is in contrast with the median age for breast cancer diagnosis in the Western population, which is 61 years, as per NIH data [24]. The median age of Indian breast cancer patients has been reported to be 50–53 years in various population-based studies carried out in different parts of the country [25]. The patient population in this study closely represents our Indian population data. Moreover, TNBC tends to present at a younger age and has been shown to be more aggressive [26].

Around 60% of our patients were in stage III. This is because, for the purposes of this study, we selected only those patients who received NACT, followed by surgery, and also the fact that patients in India have a higher stage at presentation [27]. The finding of a higher number of pCR in those who underwent BCS is explained by selection bias, as patients who had a very good clinical response were considered for BCS. So, there is a higher chance of finding a pCR in BCS patients.

In our study, tumour PDL1 expression was seen in 31% of the patients. In the published literature, the rate has varied from 40% to 50% [28, 29]. In another study, 50% of stromal cells had some PDL1 positivity [16]. Compared to these studies, our staining percentage is much lower. This may be due to the fact that most studies have combined staining of both tumour cells as well as stromal cells, while we restricted our analysis to only tumour cells. This result may also be due to geographic variation, as none of the previous studies included Indian patients. The standard regimen has been six cycles of FEC-T. With such a regimen, around 33% of our patients achieved pCR. This is consistent with global data, where pCR rates of TNBC varies from 30% to 40% [30–32].

If we analyse the role of PDL1 expression with pCR, the relationship between PDL1 expression and pCR is not clearly defined in the literature. In one study, PDL1 expression was associated with an inferior response rate [33]. However, it was a heterogeneous population of all subtypes and not specific for TNBC. In our study, the achievement of pCR did not correlate with the PDL1 expression. One possible explanation for this may be the fact that we used a standard chemotherapy regimen and did not use ICIs. It is possible that the addition of ICIs with chemotherapy might have increased the pCR rate in those who have a higher PDL1 expression.

However, if we look at the survival outcomes, both the RFS and OS were significantly better in patients who achieved a pCR. This is consistent with the well-established fact that the achievement of pCR is a strong surrogate marker for improved survival in breast cancer [11, 32]. We do agree that this is a retrospective study and the groups may not be comparable, unlike a properly conducted prospective randomised trial.

The PDL1 expression also showed a correlation with survival. For RFS, the improvement was in the outcome of borderline significance, but for OS, it was highly significant. Many other studies have also shown that PDL1 expression is associated with favourable outcomes [16, 28]. Even those who have been treated with ICIs had a better outcome with PDL1 expression [34]. The reason for RFS not reaching statistical significance may be because of the small sample size. But definitely the trend is towards better RFS, as the hazard ratio is 0.43 (0.16–1.14). On the contrary, some studies have shown inferior outcomes with PDL1 expression [35]. We feel that our data is more aligned to those of Li *et al* [16] and Beckers *et al* [28]. The reason for the difference in the outcomes compared to Asano *et al* [33] might be because of the fact that they included all comers. But our study is unique, as it only includes TNBC. So, our data might be more indicative of the actual impact on survival. PDL1 expression is indicative of higher tumour infiltration with immune cells. This might have an impact on better outcomes, as shown in other studies [36].

Taking the standard cut-off for NLR and PLR, our study did not show any difference in the outcome. This is in contrast to other retrospective studies where a higher NLR and PLR predicted inferior outcomes [17, 19, 20]. So, we feel the interplay of PDL1 expression and outcome is an evolving area, and larger prospective data are required to reach a conclusion. Moreover, there can be regional differences in the median values of NLR and PLR [37].

There are few limitations of this study. Firstly, it has a small sample size, and larger numbers are needed to extrapolate the data to the population. Secondly, this is a single centre retrospective analysis. So, a selection bias is a possibility.

## Conclusion

Overall, this study is one of the first to look at a very homogenous population of TNBC who have received standard NACT and evaluated the outcomes and response rates based on the PDL1 status. Our study proves that PDL1 expression may not correlate the response rate to NACT, but its expression may improve long-term outcomes. This study also reconfirms the fact that the achievement of pCR has a significant positive impact on the long-term survival for TNBC patients.

## Authors’ contributions

*Conceptualisation*: JG, SC, SG and RA; Methodology: JG and AD; Design of the study: JG, SC, SG and RA; Data acquisition: MD, GM and JG; Validation: AD and GM; Data analysis: JG, BB and SG; Writing: JG, MC, BB and GM.

## Compliance with ethical standards

### Funding

This study was funded by the research grant from Indian Society of Medical and Paediatric Oncology (ISMPO) 2016 for best concept ward in ISMPOCON 2016, New Delhi, India

### Conflicts of interest

Author JG has received a research grant from the Indian Society of Medical and Paediatric Oncology Conference 2016 as a prize for the best research concept award, for conducting this study. The rest of the authors have declared no conflicts of interest.

### Ethical approval

This article does not contain any studies with human participants or animals performed by any of the authors.

### Informed consent

Waiver of consent was obtained from the IRB as this was a retrospective study on tissue blocks (vide: EC/TMC/86/17).

## Figures and Tables

**Table 1.** Baseline characteristics.

Baseline characteristics	*N* = 107
Age, median (range in years)	47 (range: 28–65)
Patients with BR score recorded (*n* = 95) Mean score(range)	8 (range: 6–9)
	*N* (%)
Laterality i)Left ii)Right	61 (57) 46 (43)
Menstrual status: Postmenopausal Premenopausal	59 (55.14) 48 (44.86)
Family history of malignancy: Breast cancer in first-degree relatives Other sporadic cancers	14 (13) 7 (6.6) 7 (6.6)
Clinical staging distribution: Stage I Stage II Stage III	1 (0.9) 38 (35.51) 68 (63.55)
Baseline neutrophil lymphocyte ratio, median (IQR)	2.85 (2.06–16.82)
Baseline platelet lymphocyte ratio, median (IQR)	128 (79–165)
Chemotherapy regimen FEC-T EC/FAC/EC/AC/TAC Paclitaxel carboplatin	86 (80.37) 20 (18.69) 1 (0.9)
Adjuvant radiotherapy	107 (100)
Number of NACT cycles: Six cycles Less than six cycles	98 (91.6) 9 (8.41)
Surgery done: BCS ± AD Mastectomy	52 (48.6) 55 (51.4)
Average number of lymph nodes dissected, N (range)	28 (1-37)
Average number of lymph nodes positive, N (range)	2 (0-31)
Pathological response Complete pathological response Residual disease	35 (32.71) 72 (67.29)
PDL1 any score: Negative Positive (>1%) Uninterpretable	68 (68.69) 31 (31.31) 8

**Table 2.** Univariate logistic regression of pCR with baseline clinico-pathological parameters.

Variable (subcatergories)	*p*-value	Odds ratio (95% CI)
PDL1 score (negative versus >1%)	0.2	0.59 (0.23–1.39)
Clinical stage (I versus II versus III)	0.2	1.61 (0.73–3.50)
Number of chemo cycles (six versus less than six)	0.48	1.77 (0.34–9.03)
Type of surgery (BCS versus MRM)	<0.001	0.33 (2.08–12.5)
Menopausal status (pre versus post)	0.59	1.24 ( 0.55–2.80)
NLR (less versus more than 3)	0.2	0.6 (0.27–1.32)
PLR (less versus more than 185)	0.9	0.98 (0.33–2.88)

**Table 3.** Multivariate logistic regression of pCR with baseline clinico-pathological parameters.

Variable	*p*-value	Odd ratio (95% CI)
PDL1 score (negative versus >1%)	0.2	0.54 (0.02–1.43)
Clinical stage (I versus II versus III)	0.4	1.35 (0.56–3.22)
Number of chemo cycles (six versus less than six)	0.38	2.87 (0.26–30.73)
Type of surgery (BCS versus MRM)	0.02	3.11 (1.1–8.5)
Menopausal status (pre versus post)	0.98	1.01 (0.40–2.48)
NLR (less versus more than 3)	0.1	0.5 (0.21–1.32)
PLR (less versus more than 185)	0.8	1.1 (0.32–3.9)

**Table 4.** Cox proportional-hazards for survival outcomes.

	Relapse-free survival		Overall survival	
Variable	HR (95% CI)	*p* value	HR (95% CI)	*p* value
pCR yes pCR no	1 3.34 (1.19–9.34)	0.02	1 18.1 (2.72–146.0)	0.006
PDL1 score negative more than 1%	1 0.43 (0.16–1.14)	0.09	1 0.21 (0.06–0.75)	0.02
NLR More than 3 less than 3	1 1.16 (0.5–2.5)	0.6	1 0.89 (0.42–1.89)	0.77
PLR Less than 185 More than 185	1 0.52 (0.15–1.77)	0.2	1 0.33 (0.08–1.43)	0.14
Menopausal status: Premenopausal Postmenopausal	1 1.01 (0.49–2.1)	0.2	1 0.91 (0.38–2.18)	0.8
Clinical stage I and II III	1 1.15 (0.5–2.30)	0.5	1 1.13 (0.5–2.43)	0.3
Number of chemo cycles Less than six cycles Six cycles	1 0.96 (0.21–4.13)	0.9	1 1.48 (0.31–7.07)	0.6
Types of surgery BCS MRM	1 1.6 (0.6–3.75)	0.2	1 2.62 (0.94–7.26)	0.06
